# Ability to detect potentially inappropriate prescribing in older patients: comparative analysis between PIM-Check and STOPP/STARTv2

**DOI:** 10.1007/s00228-021-03171-4

**Published:** 2021-06-30

**Authors:** Akram Farhat, Alice Panchaud, Amal Al-Hajje, Pierre-Olivier Lang, Chantal Csajka

**Affiliations:** 1 Center for Research and Innovation in Clinical Pharmaceutical Sciences, Lausanne University Hospital and University of Lausanne, Lausanne, Switzerland; 2 Institute of Pharmaceutical Sciences of Western Switzerland, University of Geneva, University of Lausanne, Geneva, Switzerland; 3 School of Pharmaceutical Sciences, University of Geneva, Geneva, Switzerland; 4 Service of Pharmacy, Lausanne University Hospital, Lausanne, Switzerland; 5 Clinical and Epidemiological Research Laboratory, Faculty of Pharmacy, Lebanese University, Hadat, Lebanon; 6 Genolier Clinic, Genolier, Switzerland

**Keywords:** Potentially inappropriate medication list, Prescribing errors, Medication review, Aged, PIM-Check

## Abstract

**Purpose:**

Potentially inappropriate prescribing (PIP) is a source of preventable adverse drug events. The objective of this study was a comparative analysis (quantitative and qualitative) between two tools used to detect PIP, PIM-Check and STOPP/START.

**Methods:**

First, a qualitative analysis (QAC) was conducted to evaluate the concordance between the criteria, which constitute PIM-Check and the gold standard STOPP/START. Second, a retrospective comparative and observational study was performed on the list of treatment at the admission of 50 older patients hospitalized in an acute geriatric ward of a university hospital in Switzerland in 2016 using both tools.

**Results:**

The QAC has shown that 50% (57 criteria) of STOPP/START criteria are fully or partially concordant with those of PIM-Check. The retrospective study was performed on 50 patients aged 87 years, suffering from 5 co-morbidities (min–max 1–11) and treated by of 8 drugs (min–max 2–16), as medians. The prevalence of the detected PIP was 80% by PIM-Check and 90% by STOPP/START. Medication review shows that 4.2 PIP per patient were detected by PIM-Check and 3.5 PIP by STOPP/START among which 1.9 PIP was commonly detected by both tools, as means. PIM-Check detected more PIP related to cardiology, angiology, nephrology, and endocrinology in older patients but missed the PIP related to geriatric syndromes (e.g., fall, dementia, Alzheimer) detected by STOPP/START.

**Conclusions:**

By using PIM-Check in geriatric settings, some PIP will not be detected. It is considered as a limitation for this tool in this frail population but brings a certain complementarity in other areas of therapy not covered by STOPP/START.

## Introduction

Potentially inappropriate prescribing (PIP) involves the use of medications where the risk of adverse drug events (ADEs) surpasses the clinical benefits, especially when safer or more effective alternatives are available [1]. PIP also includes the use of drugs that increase the likelihood of drug-drug and drug-disease interactions, the mis-prescribing of drugs (e.g., incorrect dose, frequency and duration), and the over-use and the under-use of clinically indicated medicines (i.e., prescribing omission) [2]. PIP are highly prevalent in older patients and have been associated with preventable ADEs, hospitalization, institutional admission, death, and resource wastage [2–6]. With increasing proportions of older people worldwide, [7] quality and safety of prescribing are becoming a major public health issue [8–10].

One of the effective ways to limit PIP is medication review through the use of sets of explicit criteria [2, 11], which are lists of drugs to be avoided or introduced, based on a consensus of experts, in order to support improvements of the therapy [2]. Thus, many prescription-screening checklists, such as the Beers criteria [12], and the STOPP/START (Screening Tool of Older Persons’ Prescriptions/Screening Tool to Alert to Right Treatment) criteria [13], have been designed and validated to detect PIP in geriatric patients. Application of STOPP/START combined with education of physicians and pharmacists has been shown to be effective in optimizing prescription and minimizing PIP in this population and is becoming the reference tool in Europe [14, 15].

Awareness of multimorbidity, polypharmacy, and PIP should not be restricted to older patients hospitalized in geriatric services only [16]. Multimorbidity and polypharmacy are independent risk factors for the occurrence of PIP, whereas age is not [17]. Geriatric checklists are usually focused on specific geriatric conditions associated with the most frequently encountered PIP in this population. Inversely, some pathologies and interventions commonly encountered in internal medicine are almost never covered by geriatric checklists (*e.g.*, obesity, infectious diseases, transplantation, renal failure, and neuropathic pain) [5], while they are also frequently present in older patients. PIM-Check is a recent-designed checklist targeting an internal medicine population designed to help in detecting a large variety of PIP. This ergonomic and electronic tool might be of interest in a geriatric setting, since older patients are also frequently encountered in internal medicine services [16]. Thus, evaluating the applicability and the clinical utility of such a non-geriatric-specific tool to the aged population is of interest.

### Aim of the study

We report the results of qualitative and quantitative analyses measuring to which extent PIM-Check could expand the scope of PIP detection in older patients compared to the STOPP/START set of criteria in a geriatric population.

## Method

### Set of explicit criteria

This study was composed by two sub-studies during which the French versions of STOPP/START v2 [18] and PIM-Check criteria [5] have been considered and, respectively, qualitatively and quantitatively compared.

STOPP/START is a set of 114 criteria organized according to relevant physiological systems and updated in 2015. STOPP comprises 80 indicators for potentially inappropriate medications (PIMs) including drug–drug and drug–disease interactions. START assesses the under-use of medicines for several common conditions simultaneously and incorporates 34 evidence-based indicators for potentially prescribing omission (PPO—when no contraindication to prescription exists). Each STOPP and START criterion is accompanied by a concise explanation as to why the prescribing practice may be inappropriate and appropriate. The tool is available and validated in English [13] and in French [18].

The PIM-Check (Potentially Inappropriate Medication checklist for Patients in Internal Medicine) electronic prescription-screening checklist [5] consists of 160 statements for pathologies and drugs commonly encountered in internal medicine, which are divided into 17 medical domains and 56 pathologies. Seventy-four (46%) statements are related to under-prescription (PPO), 36 (23%) to over-prescription (PIMs), 16 (10%) to drug-drug interactions (DDI) and 34 (21%) to other PIP (OTH) (*e.g.*, insufficient drug monitoring, dose adjustment, choice of medication). Rationales for the statements are provided along with 233 references, 116 recommendations (e.g., dose adjustment, alternatives and monitoring), 93 remarks (*e.g.*, definitions, reminders and useful lists of drugs) and 24 useful web links. The tool is available and validated, in French and in English (http://app.pimcheck.org/#/accueil/en).

### Qualitative analysis of concordance (QAC) of the criteria between PIM-Check and STOPP/START

For this qualitative step, the “concordance” between all the 160 and 114 criteria composing PIM-Check and STOPP/START, respectively, was compared with the aim to map the field of detection of PIP situations globally and specifically investigated by the two tools. All criteria composing the two detection tools were thus classified into three groups based on the medication and the clinical conditions in which it may be applicable: fully concordant (criteria present in both tools and cover the same clinical conditions), partially concordant (criteria present in both tools, but which may be applicable differently depending on the clinical situation (for example STOPP recommend to avoid metformin if eGFR ˂30 ml/min vs PIM-Check recommend to withhold metformin in hospitalized diabetic patients in unstable conditions, in case of surgery, or in case of the injection of an iodine contrast product, particularly with polymorbidity or renal failure)), and non-concordant (present only in one tool). Five experts (*i.e.*, 1 geriatrician and 4 clinical pharmacists) participated in this process analysis. The consensus was reached when all the five experts agreed. Disagreements were resolved through discussion.

### Quantitative retrospective study

For the quantitative analysis, the capacity of the two tools to detect PIP (PIMs and PPO) was investigated and compared. Thus, the two tools were used by a clinical pharmacist to conduct a medication review for patients consecutively hospitalized, between July 1 and 31, 2016 in the Acute Care for Elders (ACE) unit of the University Hospital of Lausanne in Switzerland. In this unit were hospitalized patients aged 65 years or over, with one geriatric syndrome or more and requiring acute medical care. Thus, in priority were admitted patients with gait disturbances and/or having fallen at least once in the current/past year, with delirium, cognitive impairment, malnutrition and/or with multimorbidity. The criteria for non-admission to the ACE were patients with instable medical condition that might require continuous/intensive care within 24 h and/or admission to a psychiatric ward (*e.g.*, because of a high risk of suicide, a runaway and/or violent patient).

The datasets compiled retrospectively from chart review for each patient, included information on sociodemographic characteristics, medications, co-morbidities and laboratory results. Drugs were classified using the Anatomical Therapeutic Chemical (ATC) Classification System [19]. Polypharmacy was defined as 5 or more chronic daily medications; hyper-polypharmacy was defined as 10 or more [20].

To identify instances of PIMs and PPO, a medication review was conducted by a clinical pharmacist using STOPP/START and PIM-Check. All instances of PIMs and PPO thus identified were respectively described by the related specific criteria.

Applicable PIP were recorded and classified according to the type of PIP detected (PIMs/STOPP, PPO/START, DDI and OTH), as well as the physiopathological classes. Some PIP were considered as inapplicable because some elements of the criteria were not relevant for the patient. This typically concerns criteria comprising minimal cut-offs of laboratory values (hemoglobin or renal function values) that the patient does not reach (*e.g.*, erythropoiesis-stimulating agents (ESA) in chronic kidney disease (CKD) at the hemoglobin level < 10 g/dl). These false positive as well as duplicates (*e.g.*, prescription of a treatment already suggested by another criteria) were eliminated.

The qualitative analysis on the “detected PIP” was conducted on the basis of the results of the qualitative study. Thus, for each PIP detected using PIM-Check, the concordant criteria were matched with the corresponding PIP detected by STOP/START and vice versa. Results were then classified into three different groups as follows: PIP detected by both tools, PIP detected only by PIM-Check and PIP detected only by STOPP/START. For each group, the proportion of PIP with their level of concordance (fully concordant, partially concordant and non-concordant criteria) was presented.

### Statistical analysis

For the qualitative analysis, descriptive statistics were considered to present all the criteria according to their concordance level (i.e., fully concordant, partially concordant and non-concordant). In the quantitative retrospective study, a descriptive analysis of the patients’ socio-demographic and clinical characteristics was performed as well as for each type of PIP detected (PIMs/STOPP, PPO/START, DDI and OTH). Results were presented as mean with standard deviation (SD), median, min–max and/or interquartile range for quantitative data; proportion with percentage was given for qualitative data. In addition to the numbers of PIP detected by each tool, the mean duration of the medication review was also compared using a Student *t* test (paired sample *t* test). PIP were presented as classified according to the physiological system and according to the tool that detected them (PIM-Check, STOPP/START or both) with the level of concordance (fully concordant, partially concordant and non-concordant criteria). All analyses were performed with SPSS (Statistical Package for the Social Sciences) version 23 with a significant threshold set at *p* = 0.05.

## Results

### QAC of the criteria between PIM-Check and STOPP/START

The qualitative comparison between the 160 PIM-Check and the 114 STOPP/START criteria showed that 50% (57 criteria) of STOPP/START criteria were concordant with those of PIM-Check, with only 4 couples (7%) fully concordant and 53 couples (93%) partially concordant. The partial mismatch was due to the larger scope of the clinical conditions covered by one tool within the same class of medications. Many criteria were addressed only by one tool, which cover specific clinical disease or conditions or medications. Criteria related to infectious diseases (23 criteria), addictology (9 criteria), obesity (4 criteria) and transplantation (2 criteria) were addressed only by PIM-Check, whereas falls, Alzheimer’s disease and related disorders were considered in STOPP/START only. As for drugs, only contraceptive drugs were considered non applicable to this population.

### Quantitative retrospective study

During the study period considered, 50 patients were consecutively admitted in the ACE unit. The medical datasets were retrospectively analyzed and the patients’ characteristics are presented in Table 1. The majority of patients were female (39/50) and the sample’s median age was 87 years (Inter Quartile Range (IQR): 82–92).

**Table 1:** Socio-demographic and clinical characteristics of the study population

Baseline characteristics		
Sex *n* (%) Male Female	11 39	(22) (78)
Age: median (IQR) 67–74 *n* (%) 75–84 *n* (%) 85–94 *n* (%) 95–104 *n* (%)	87 5 15 28 2	(82 – 92) (10) (30) (56) (4)
Living *n* (%) Alone (at home) with others (at home or in an institution)	33 17	(66) (34)
Number of drugs on admission: median (IQR) < 5 *n* (%) ≥ 5 *n* (%)	8 24 26	(3 – 11) (48) (52)
Most prescribed drugs (according to ATC classification): *n* (%) Nervous system Alimentary tract and metabolism Cardiovascular system Blood and blood forming organs	102 96 71 26	(27) (25.4) (18.8) (6.9)
Number of active diseases: median (IQR) < 5 *n* (%) ≥ 5 *n* (%)	5 24 26	(3 – 6) (48) (52)
Hospital stay (days): median (IQR) < 13 *n* (%) ≥ 13 *n* (%)	12.5 25 25	(7 – 18.25) (50) (50)
Most frequent co-morbidities: *n* (%) Hypertension Chronic kidney disease Dementia Heart diseases Dyslipidemia Anemia Depression Diabetes Osteoporosis Atrial fibrillation Anxiety Alzheimer	30 26 20 20 15 14 12 11 11 11 10 8	(60) (52) (40) (40) (30) (28) (24) (22) (22) (22) (20) (16)
Number of hospitalizations in the last year: median (IQR) < 2 *n* (%) ≥ 2 *n* (%)	1 31 19	(0 – 2) (62) (38)

*IQR* inter quartile range, *ATC* anatomical therapeutic chemical

At the time of admission, the 50 patients totalized 378 prescribed drugs for a median of 8 drugs per patient (IQR: 3–11). Just over half of patients (*n* = 26) were considered with polypharmacy (> 5 drugs), among which half presented hyper-polypharmacy (> 10 drugs); none were without any treatment (min–max 2–16). The most prescribed drugs according to the ATC classification system were those of the nervous system (27.0%), digestive tract and metabolism (25.4%), cardiovascular system (18.8%), and blood and blood forming organs (6.9%). On average, these 50 patients had 5 comorbid conditions (min–max 1–11). The most frequently encountered were hypertension (30/50), chronic kidney disease (26/50), cognitive disorders (20/50), heart diseases (20/50) and dyslipidemia (15/50).

When the medication review was operated, a total of 290 different PIP were identified among the 50 datasets; 209 were detected using PIM-Check (mean per patient 4.2) and 174 with STOPP/START (mean per patient 3.5). This process lasted on average 3.6 min with PIM-Check (electronic tool) vs. 9.4 min for STOPP/START (paper-checklist) (*p* < 0.01). PIM-Check detected less PIMs than STOPP (28 and 47 respectively) and more PPO than START (138 and 127 respectively). The detailed results according the type of PIP are presented in Table 2.

**Table 2:** Comparison between the number of PIP detected by PIM-Check and STOPP/START

	PIM-Check	STOPP/START	*p*-value
Total number of criteria per tool	160	114	
Number of applicable^a^ PIP	209	174	
- PIMs/STOPP	28	47	
- PPO/START	138	127	
- Related to an interaction	15	–	
- Other	28	–	
Applicable^a^ PIP mean	4.2	3.5	0.033
Mean duration of a medication review (min)	3.6	9.4	< 0.001

*PIP* Potentially Inappropriate Prescribing; *STOPP/START* Screening Tool of Older Persons’ Prescriptions/Screening Tool to Alert to Right Treatment, *PIM-Check* Potentially Inappropriate Medication checklist for Patients in Internal Medicine, *PPO* potentially prescribing omission, *PIMs* potentially inappropriate medications

^a^Applicable: pertinent PIP after the elimination of false positives and duplicates

**Fig. 1:**
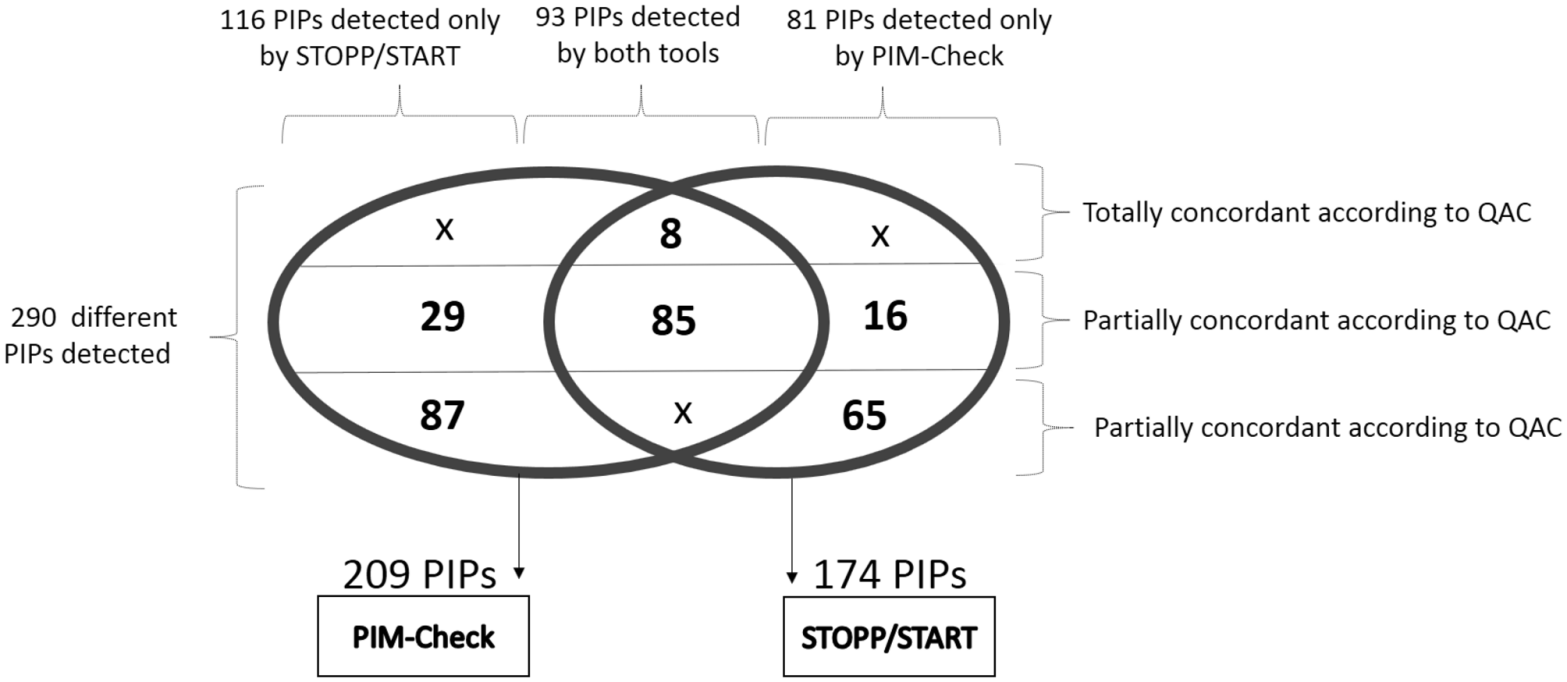
Classification of PIP according to their level of concordance. PIP potentially inappropriate prescribing, QAC qualitative analysis of concordance

Classification of PIP according to their level of concordance is shown in Fig. 1. Thus, when 93/290 PIP were detected by both tools, 116/290 were by PIM-Check and 81/290 with STOPP/START only. Among the 93 PIP conjointly detected by the two sets of criteria, 8 were fully concordant couples according to the classification elaborated during the qualitative step and 85 were partially. Of the 116 PIP detected by PIM-Check only, none were fully concordant couples, 29 were partially concordant couples and 87 classified as non-concordant criteria. Among PIP identified by STOPP/START criteria only, respectively, 16 and 65 PIP were classified as partially and non-concordant. None were found as fully concordant.

At least 1 PIP was detected in 80% (*n* = 40) of patients by PIM-Check (≥ 1 PIMs in 46% of patients, ≥ 1 PPO in 72%, ≥ 1 IAM in 20%, and ≥ 1 OTH in 40%). At least 1 PIP was detected in 90% (*n* = 45) by STOPP/START (≥ 1 STOPP in 56% of patients and ≥ 1 START in 82%, i.e., 48% had STOPP and START PIP, 8% had only STOPP PIP, and 34% had only START PIP).

PIP detected by each tool and classified by physiopathological system are presented in Table 3. The majority of PIP detected by PIM-Check were related to “cardiology” (21.5%; 45 PIP), “vaccination” (16.3%; 34 PIP) and “nephrology” (14.8%; 31 PIP). The majority of PIP detected by STOPP/START were related to “vaccination” (23.6%; 41 PIP), “musculoskeletal system” (23%; 40 PIP) and “cardiovascular system” (15.5%; 27 PIP).

**Table 3:** Comparison between the classes of PIP detected by PIM-Check and STOPP/START

	Number of criteria per tool	Applicable^a^ PIP detected		Number of criteria per Tool	Applicable^a^ PIP detected
PIM-Check criteria domain			STOPP/START criteria domain		
- Cardiology	26	45	- Cardiology (stopp + start)	21	27
- Angiology/Hemostasis	9	23	- Angiology/Hemostasis (stop)	11	4
- Pneumology	9	7	- Pneumology (stopp + start)	7	3
- Nephrology	8	31	- Nephrology (stopp)	8	6
- Gastroenterology	10	5	- Gastroenterology (stopp + start)	6	5
- Rheumatology	11	7	- Rheumatology (stopp + start)	16	40
- Neurology + Psychiatry + Ophthalmology	13	23	- Neurology and Psychiatry (stopp) + Neurology and Ophthalmology (start)	20	24
- Pain and Analgesia	8	6	- Pain and Analgesia (stopp + start)	5	2
- Endocrinology	16	21	- Endocrinology (stopp + start)	7	1
- Vaccination	4	34	- Vaccination (start)	2	41
- Infectiology	23	1			
- Dependencies	9	0			
- Obesity	4	0			
- Pharmacology and Toxicology	8	6			
- Transplants	2	0			
			- Indication of treatment (stopp)	3	5
			- Urogenital system (start)	3	6
			- Drugs associated with increased risk of fall (stopp)	4	10
			- Anticholinergic medications (stopp)	1	0
Total	160	209		114	174

*PIP* potentially inappropriate prescribing, *STOPP/START* Screening Tool of Older Persons’ Prescriptions/Screening Tool to Alert to Right Treatment, *PIM-Check* Potentially Inappropriate Medication checklist for Patients in Internal Medicine

^a^Applicable: Pertinent PIP after the elimination of false positives and duplicates

The comparison of classified detected PIP (Table 3) shows that PIM-Check identified more PIP than STOPP/START in several pharmacotherapy areas (cardiology, angiology/hemostasis, nephrology and endocrinology). The qualitative description of the identified difference in the criteria is presented in Table 4.

**Table 4:** Qualitative comparison between PIP detected by PIM-Check and STOPP/START

Domain	Difference	Detected PIP
Cardiology	PIM-Check identified 18 more PIP	PIM-Check recommended statins (14 among the 18 additional PIP detected) in 5 patients with dyslipidemia and 9 patients with high cardiovascular risk or suffering from stable ischemic heart disease aged > 85 years
		PIM-Check suggested ACEI or ARBs prescription (4 among the 18 additional PIP detected) in patients with diabetes or CKD
Angiology/hemostasis	PIM-Check identified 19 more PIP	PIM-Check recommended a prophylactic anticoagulation (9 among the 19 additional PIP detected) in hospitalized patients with risk of thrombosis
		PIM-Check recommended patient education (10 among the 19 additional PIP detected) in patients receiving oral anticoagulation
Nephrology	PIM-Check identified 29 more PIP	PIM-Check recommended a prescription of calcium and vitamin D (12 among the 29 additional PIP detected), as well as a prescription of phosphate-binding agents (12 among the 29 additional PIP detected) in patients with CKD
		PIM-Check recommended ESA (4 among the 29 additional PIP detected) in CKD patients with a Hb level < 10 g/dL
		PIM-Check recommended dose adjustment (1 among the 29 additional PIP detected) in patient with CKD
	STOPP identified 4 more PIP	STOPP/START recommended to stop antimuscarinic drugs in patients with dementia (4 among the 4 additional PIP detected)
Endocrinology	PIM-Check identified 20 more PIP	PIM-Check recommended the prescription of metformin as a first-line treatment (6 among the 20 additional PIP detected), as well as the adjustment of the antidiabetic therapy according to HbA1c targets (5 among the 20 additional PIP detected) in patients with diabetes
		PIM-Check recommended a prescription of statins (3 among the 20 additional PIP detected) and aspirin (2 among the 20 additional PIP detected) in patients with diabetes and high cardiovascular risk
		PIM-Check suggested to withhold metformin (3 among the 20 of the remaining additional PIP) in patients with moderate CKD
		The 1 remaining additional PIP detected by PIM-Check was also detected by STOPP/START but classified in the nephrology domain (i.e., to stop metformin in patients with a GFR < 30 ml/min in STOPP/START)
Musculoskeletal system	START identified 33 more PIP	STOPP/START recommended vitamin D supplement alone (15 among the 33 additional PIP detected) in patients with falls, as well as bone anti-resorptive or anabolic therapy (10 among the 33 additional PIP detected), and vitamin D and calcium supplements (8 among the 33 remaining additional PIP detected) in patients with osteoporosis
Vaccination	START identified 7 more PIP	STOPP/START recommended to perform an influenza vaccine during the flu season (7 among 7 PIP detected) in patents without risk factors for complications other than age > 65 years
Pharmacology and Toxicology	PIM-Check detected 6 PIP	
Drugs associated with increased risk of fall	STOPP/START detected 10 PIP	
Non-indicated treatments	STOPP/START detected 5 PIP	
Drugs associated to urogenital system	STOPP/START detected 10 PIP	

*PIP* potentially inappropriate prescribing, *PIM*-Check Potentially Inappropriate Medication checklist for Patients in Internal Medicine, *STOPP/START* Screening Tool of Older Persons’ Prescriptions/Screening Tool to Alert to Right Treatment, *CKD* chronic kidney disease, *ESA* erythropoiesis-stimulating agents

## Discussion

This study is the first analysis qualitatively and quantitatively comparing the scope of PIP detection of STOPP/START and PIM-Check set of criteria and the usefulness of PIM-Check in a geriatric population. This study has identified some major differences in their potential use in acutely ill vulnerable and older hospitalized patients. The application of both tools on our population of older patients indicates that the prevalence of PIP was greater with STOPP/START than with PIM-Check (90% vs 80% of patients), although STOPP/START detected a lower number of PIP (174 vs 209). With a low overlap between the two tools, PIM-Check detected mostly PIP related to common medical conditions and failed in detecting those linked with geriatric syndromes (*e.g.*, falls, Alzheimer’s disease and associated disorders) frequent in geriatric medicine.

PIM-Check was originally designed for patients hospitalized in internal medicine comprising a population with a wider range of age. The current available literature evaluating the use of this tool is composed by three studies [21–23] conducted in internal medicine settings. The first study [21] is the only one performing a comparison between PIM-Check and STOPP/START. In this study, the study population was slightly younger (mean age 77 years), with however 84% of patients aged > 65 years [21], with a mean number of drugs close to our findings (7 drugs per patient). The number of PIP per patient detected by PIM-Check was higher in comparison to our study (6.1 vs 4.2). This difference could be explained by difference in dealing with duplicates (*i.e.*, two or more criteria detecting the same inappropriate prescription) between both studies. As expected, STOPP/START detected a lower number of PIP per patient in this study compared to our study (2.2 *vs* 3.5), reflecting the potential lower rate of patients with geriatric syndromes. With respect to the speed processing of the medication review, this study similarly reported the benefit of using PIM-Check (4 *vs.* 10 min for STOPP/START). However, this study did not address qualitative aspects in the comparison between the two tools such as the scope of the PIP they detected. In the second study [22], PIM-Check was used to measure PIP in 45–75-year-old patients hospitalized in internal medicine, respiratory medicine and cardiology. In a sub-group of older individuals, in average, 0.7 PIP per patient was identified, considerably lower than that measured in the present study despite a mean number of drugs of 10.4 drugs per patient. This discrepancy probably found its origin in the medical profile of the population studied in which the prevalence of comorbid conditions was much lower than our geriatric population. The last study [23] has focused on the effect of using PIM-Check on detecting and reducing PIP. Compared to controls (*i.e.*, standard care), PIM-Check used by physicians did not significantly reduce the mean number of PIP per patient among internal medicine patients, which could be related to the low percentage of acceptance of recommendations by physicians. As for STOPP/START, a few prospective studies have evaluated its effectiveness in detecting PIP. A prospective study conducted in 900 patients aged ≥ 65 years and admitted to acute geriatric units in six European hospitals showed similar results concerning high polypharmacy (58%) with PIMs ranging from 34.7 to 77.3%, in line with our results (56% by STOPP and 46% by PIM-Check) [24]. The prevalence of PPO (51.3 to 72.7% START) was slightly lower than that in our observation (82% by START and 72% by PIM-Check).

The results of the qualitative step of the present study identified that PIM-Check targeted preferentially PIP related to PPO, DDI and therapeutic follow-ups, and thus would tend to recommend treatment initiation. The set of STOPP/START criteria mainly focused on PIP associated with PIMs, drug comorbidity and drug-geriatric syndrome interactions, and thus would favor deprescribing. However, the retrospective study shows that both tools have rather contributed to initiate missing treatments than deprescribing potentially inappropriate ones, and were mostly linked to five criteria related to vaccination and rheumatology sections of START, and to four criteria of vaccination and nephrology section of PIM-Check. Unsurprisingly, frequent treatments in geriatrics such as influenza and pneumococcal immunization, calcium and vitamin D supplementation, bisphosphonates and phosphate-binding agents were among the most frequently PPO. While influenza and pneumococcal immunization were commonly recommended for all aged patients by both tools (totally concordant), calcium and vitamin D were not (partially concordant). Thus, bisphosphonates were recommended only by START and phosphate-binding agents by PIM-Check (non-concordant).

PIM-Check detected many PIP that were not necessarily suitable to geriatric patients and failed to detect potentially drug-geriatric syndrome interactions. For example, a statin is recommended in all patients with hypercholesterolemia, high cardiovascular risk with or without confirmed vascular complication by PIM-Check, whereas some limitations are present in the START criteria (only as secondary prevention with appraisal of frailty and/or functional status, life expectancy, comorbidities, and polypharmacy). Similarly, PIM-Check did not recommend a systematic introduction of calcium and vitamin D supplementation, and bisphosphonate therapy in patients with osteoporosis, nor vitamin D supplementation in fallers and patients with osteopenia as systematically suggested by START. More importantly, PIM-Check failed to recommend deprescribing drugs with anticholinergic effect in those with cognitive impairment or narrow-angle glaucoma for example.

Inversely, STOPP/START missed the detection of some PIP frequently encountered in internal medicine patients, such as, ACEI or ARB in diabetic patients to control hypertension and/or microalbuminuria; systematic prophylactic anticoagulation therapy in bedridden patients; or calcium, vitamin D and phosphate-binding agents in patients with severe CKD. STOPP/START also failed to recommend patients' education for those receiving oral anticoagulant therapy and/or specific adjustment of antidiabetic therapy according to HbA1c targets.

This study suffered from some limitations. First, the relatively small sample size available for this study did not allow to explore all possible discrepancies between the two tools. Second, the retrospective and observational nature of the study did not allow to follow up and compare the clinical impact of PIP detected by each tool which will be the main objective of an ongoing randomized controlled trial conducted in the same setting (ClinicalTrials.gov ID: NCT04028583).

## Conclusion

Balanced and safe prescribing is difficult to achieve especially in older adults with multiple diseases and treatments. Using PIM-Check in the geriatric setting can expose clinicians to miss some PIP related to geriatric syndromes, which is considered as a limitation for this tool in this frail population. On the other hand, PIM-Check detected more PIP related to cardiology, angiology, nephrology and endocrinology, which may bring a certain complementarity to the current gold standard STOPP/START. To further increase the detection of PIP and thus the optimization of pharmacotherapy in geriatrics, the development of a combined tool might be a next step.
